# Supplementary material for: Multi‐dataset identification of innovative feature genes and molecular mechanisms in keratoconus
Source: J Cell Mol Med. 2024 Sep 19;28(18):e70079. doi: 10.1111/jcmm.70079 (PMC11412914; doi:10.1111/jcmm.70079)
Supplement: Supplementary file 2 — Material S2. [file JCMM-28-e70079-s002.zip › 7.GSEA analysis/3.DBNDD1/KEGG_OLFACTORY_TRANSDUCTION.html]

Details for gene set KEGG\_OLFACTORY\_TRANSDUCTION[GSEA]

|  || Dataset | DBNDD1.group.cls#h\_versus\_l |
| Phenotype | group.cls#h\_versus\_l |
| Upregulated in class | h |
| GeneSet | KEGG\_OLFACTORY\_TRANSDUCTION |
| Enrichment Score (ES) | 0.245256 |
| Normalized Enrichment Score (NES) | 0.8148634 |
| Nominal p-value | 0.9747748 |
| FDR q-value | 1.0 |
| FWER p-Value | 1.0 |
Table: GSEA Results Summary

  

Fig 1: Enrichment plot: KEGG\_OLFACTORY\_TRANSDUCTION      
 Profile of the Running ES Score & Positions of GeneSet Members on the Rank Ordered List

  

| SYMBOL | TITLE | RANK IN GENE LIST | RANK METRIC SCORE | RUNNING ES | CORE ENRICHMENT || 1 | OR52E8 | na | 43 | 0.689 | 0.0095 | Yes |
| 2 | OR2T6 | na | 149 | 0.572 | 0.0153 | Yes |
| 3 | OR13D1 | na | 183 | 0.553 | 0.0229 | Yes |
| 4 | CAMK2B | na | 217 | 0.536 | 0.0303 | Yes |
| 5 | OR8J1 | na | 366 | 0.485 | 0.0335 | Yes |
| 6 | OR2AG1 | na | 449 | 0.465 | 0.0384 | Yes |
| 7 | OR4Q3 | na | 509 | 0.450 | 0.0436 | Yes |
| 8 | OR4C6 | na | 573 | 0.433 | 0.0485 | Yes |
| 9 | OR9I1 | na | 1043 | 0.377 | 0.0407 | Yes |
| 10 | OR7A17 | na | 1097 | 0.372 | 0.0450 | Yes |
| 11 | OR4K15 | na | 1126 | 0.370 | 0.0499 | Yes |
| 12 | OR1K1 | na | 1129 | 0.369 | 0.0556 | Yes |
| 13 | OR5T3 | na | 1177 | 0.364 | 0.0599 | Yes |
| 14 | OR4K17 | na | 1178 | 0.364 | 0.0655 | Yes |
| 15 | OR13C4 | na | 1321 | 0.351 | 0.0669 | Yes |
| 16 | OR10A4 | na | 1430 | 0.343 | 0.0690 | Yes |
| 17 | OR51A7 | na | 1587 | 0.329 | 0.0696 | Yes |
| 18 | CLCA1 | na | 1758 | 0.316 | 0.0696 | Yes |
| 19 | OR4N4 | na | 1800 | 0.313 | 0.0733 | Yes |
| 20 | OR8S1 | na | 1864 | 0.309 | 0.0762 | Yes |
| 21 | OR8D1 | na | 1875 | 0.308 | 0.0807 | Yes |
| 22 | OR14I1 | na | 1935 | 0.304 | 0.0837 | Yes |
| 23 | OR2G2 | na | 1937 | 0.304 | 0.0884 | Yes |
| 24 | OR4D1 | na | 1953 | 0.303 | 0.0927 | Yes |
| 25 | OR5M8 | na | 1974 | 0.301 | 0.0968 | Yes |
| 26 | OR10K2 | na | 2065 | 0.295 | 0.0988 | Yes |
| 27 | OR10T2 | na | 2123 | 0.291 | 0.1016 | Yes |
| 28 | OR6V1 | na | 2132 | 0.291 | 0.1059 | Yes |
| 29 | OR12D3 | na | 2184 | 0.288 | 0.1089 | Yes |
| 30 | OR2A12 | na | 2255 | 0.284 | 0.1113 | Yes |
| 31 | OR9K2 | na | 2302 | 0.281 | 0.1143 | Yes |
| 32 | OR10H1 | na | 2360 | 0.278 | 0.1170 | Yes |
| 33 | OR10A2 | na | 2457 | 0.274 | 0.1185 | Yes |
| 34 | OR5AR1 | na | 2545 | 0.270 | 0.1201 | Yes |
| 35 | OR7C1 | na | 2560 | 0.270 | 0.1239 | Yes |
| 36 | OR2M4 | na | 2575 | 0.269 | 0.1277 | Yes |
| 37 | OR10A3 | na | 2634 | 0.266 | 0.1301 | Yes |
| 38 | OR2AT4 | na | 2659 | 0.265 | 0.1336 | Yes |
| 39 | OR13C3 | na | 2663 | 0.265 | 0.1376 | Yes |
| 40 | OR56B4 | na | 2666 | 0.265 | 0.1417 | Yes |
| 41 | OR52E6 | na | 2692 | 0.264 | 0.1450 | Yes |
| 42 | OR2A2 | na | 2783 | 0.260 | 0.1465 | Yes |
| 43 | OR5B3 | na | 2797 | 0.260 | 0.1501 | Yes |
| 44 | OR52N1 | na | 2876 | 0.256 | 0.1518 | Yes |
| 45 | OR6C65 | na | 2930 | 0.255 | 0.1542 | Yes |
| 46 | OR4S2 | na | 3022 | 0.251 | 0.1555 | Yes |
| 47 | OR6B2 | na | 3038 | 0.250 | 0.1589 | Yes |
| 48 | OR10AG1 | na | 3061 | 0.249 | 0.1622 | Yes |
| 49 | OR14C36 | na | 3154 | 0.245 | 0.1633 | Yes |
| 50 | OR9Q1 | na | 3190 | 0.244 | 0.1661 | Yes |
| 51 | OR5AS1 | na | 3240 | 0.242 | 0.1684 | Yes |
| 52 | OR52B6 | na | 3339 | 0.238 | 0.1693 | Yes |
| 53 | OR5T1 | na | 3357 | 0.237 | 0.1724 | Yes |
| 54 | OR3A1 | na | 3443 | 0.234 | 0.1736 | Yes |
| 55 | CNGA3 | na | 3522 | 0.231 | 0.1749 | Yes |
| 56 | OR14A16 | na | 3639 | 0.226 | 0.1751 | Yes |
| 57 | OR52B2 | na | 3643 | 0.226 | 0.1785 | Yes |
| 58 | OR5M1 | na | 3679 | 0.224 | 0.1809 | Yes |
| 59 | OR10V1 | na | 3689 | 0.224 | 0.1842 | Yes |
| 60 | OR52H1 | na | 3691 | 0.224 | 0.1876 | Yes |
| 61 | OR6C1 | na | 3733 | 0.223 | 0.1899 | Yes |
| 62 | OR5C1 | na | 3753 | 0.222 | 0.1928 | Yes |
| 63 | OR5F1 | na | 3778 | 0.221 | 0.1955 | Yes |
| 64 | OR13C9 | na | 3843 | 0.218 | 0.1970 | Yes |
| 65 | OR5D13 | na | 3846 | 0.218 | 0.2004 | Yes |
| 66 | OR2T34 | na | 3883 | 0.217 | 0.2027 | Yes |
| 67 | OR10A7 | na | 3974 | 0.214 | 0.2034 | Yes |
| 68 | CAMK2A | na | 3995 | 0.213 | 0.2062 | Yes |
| 69 | OR5B17 | na | 4014 | 0.213 | 0.2089 | Yes |
| 70 | OR12D2 | na | 4035 | 0.213 | 0.2117 | Yes |
| 71 | OR10X1 | na | 4066 | 0.211 | 0.2141 | Yes |
| 72 | OR8B4 | na | 4136 | 0.209 | 0.2153 | Yes |
| 73 | OR10J1 | na | 4160 | 0.208 | 0.2179 | Yes |
| 74 | OR5H2 | na | 4180 | 0.208 | 0.2206 | Yes |
| 75 | OR52N5 | na | 4194 | 0.207 | 0.2234 | Yes |
| 76 | OR6C2 | na | 4220 | 0.206 | 0.2259 | Yes |
| 77 | OR10S1 | na | 4251 | 0.205 | 0.2282 | Yes |
| 78 | CALML6 | na | 4335 | 0.202 | 0.2289 | Yes |
| 79 | OR8D2 | na | 4337 | 0.202 | 0.2320 | Yes |
| 80 | OR2T1 | na | 4409 | 0.200 | 0.2331 | Yes |
| 81 | OR1N1 | na | 4418 | 0.199 | 0.2359 | Yes |
| 82 | OR4A16 | na | 4455 | 0.198 | 0.2380 | Yes |
| 83 | OR1D5 | na | 4619 | 0.193 | 0.2362 | Yes |
| 84 | OR5D18 | na | 4623 | 0.192 | 0.2391 | Yes |
| 85 | OR8B12 | na | 4743 | 0.188 | 0.2386 | Yes |
| 86 | OR2L3 | na | 4795 | 0.187 | 0.2400 | Yes |
| 87 | PRKG2 | na | 4906 | 0.183 | 0.2396 | Yes |
| 88 | OR10G8 | na | 4996 | 0.181 | 0.2399 | Yes |
| 89 | OR51F2 | na | 5032 | 0.180 | 0.2416 | Yes |
| 90 | OR51Q1 | na | 5150 | 0.175 | 0.2409 | Yes |
| 91 | OR4P4 | na | 5311 | 0.170 | 0.2389 | Yes |
| 92 | OR6C6 | na | 5397 | 0.168 | 0.2391 | Yes |
| 93 | OR2T2 | na | 5439 | 0.166 | 0.2404 | Yes |
| 94 | OR2C3 | na | 5508 | 0.164 | 0.2410 | Yes |
| 95 | OR2T11 | na | 5617 | 0.161 | 0.2404 | Yes |
| 96 | OR4F6 | na | 5618 | 0.161 | 0.2429 | Yes |
| 97 | OR11G2 | na | 5623 | 0.161 | 0.2453 | Yes |
| 98 | OR2T8 | na | 5721 | 0.158 | 0.2449 | No |
| 99 | OR6C75 | na | 5983 | 0.151 | 0.2396 | No |
| 100 | OR5D16 | na | 5988 | 0.151 | 0.2419 | No |
| 101 | OR7A5 | na | 6068 | 0.149 | 0.2419 | No |
| 102 | OR2W3 | na | 6100 | 0.148 | 0.2433 | No |
| 103 | OR2A14 | na | 6115 | 0.147 | 0.2452 | No |
| 104 | OR4A47 | na | 6324 | 0.142 | 0.2413 | No |
| 105 | OR10G3 | na | 6525 | 0.136 | 0.2376 | No |
| 106 | OR56A5 | na | 6658 | 0.132 | 0.2358 | No |
| 107 | OR2T10 | na | 7097 | 0.122 | 0.2250 | No |
| 108 | OR9A4 | na | 7175 | 0.120 | 0.2246 | No |
| 109 | OR4C46 | na | 7350 | 0.117 | 0.2213 | No |
| 110 | OR10H4 | na | 7444 | 0.115 | 0.2204 | No |
| 111 | OR1B1 | na | 7569 | 0.112 | 0.2185 | No |
| 112 | OR2M7 | na | 7621 | 0.111 | 0.2188 | No |
| 113 | OR6N1 | na | 7667 | 0.111 | 0.2192 | No |
| 114 | OR6K3 | na | 7734 | 0.109 | 0.2190 | No |
| 115 | CALML5 | na | 7930 | 0.105 | 0.2149 | No |
| 116 | OR56A1 | na | 8029 | 0.103 | 0.2137 | No |
| 117 | OR52I1 | na | 8106 | 0.101 | 0.2130 | No |
| 118 | OR1L1 | na | 8204 | 0.100 | 0.2118 | No |
| 119 | OR2L2 | na | 8234 | 0.099 | 0.2125 | No |
| 120 | OR8G5 | na | 8264 | 0.099 | 0.2132 | No |
| 121 | OR6C74 | na | 8490 | 0.095 | 0.2081 | No |
| 122 | OR6K6 | na | 8646 | 0.092 | 0.2050 | No |
| 123 | OR10P1 | na | 8711 | 0.091 | 0.2046 | No |
| 124 | OR2B2 | na | 8895 | 0.087 | 0.2006 | No |
| 125 | OR5B21 | na | 8896 | 0.087 | 0.2019 | No |
| 126 | OR1A2 | na | 8955 | 0.086 | 0.2016 | No |
| 127 | OR4C11 | na | 9130 | 0.084 | 0.1978 | No |
| 128 | OR5AN1 | na | 9210 | 0.082 | 0.1968 | No |
| 129 | OR4C3 | na | 9240 | 0.082 | 0.1972 | No |
| 130 | OR13F1 | na | 9286 | 0.081 | 0.1972 | No |
| 131 | OR5D14 | na | 9409 | 0.080 | 0.1949 | No |
| 132 | OR5L2 | na | 9423 | 0.079 | 0.1957 | No |
| 133 | OR8K1 | na | 9514 | 0.078 | 0.1943 | No |
| 134 | OR3A2 | na | 9803 | 0.074 | 0.1871 | No |
| 135 | OR6N2 | na | 9896 | 0.073 | 0.1856 | No |
| 136 | OR1L8 | na | 10138 | 0.070 | 0.1796 | No |
| 137 | OR5K2 | na | 10155 | 0.069 | 0.1802 | No |
| 138 | CALML3 | na | 10231 | 0.068 | 0.1791 | No |
| 139 | OR5P3 | na | 10571 | 0.064 | 0.1702 | No |
| 140 | OR52K2 | na | 10743 | 0.061 | 0.1662 | No |
| 141 | OR6A2 | na | 10827 | 0.060 | 0.1647 | No |
| 142 | OR51E1 | na | 10906 | 0.059 | 0.1634 | No |
| 143 | OR6M1 | na | 10932 | 0.059 | 0.1636 | No |
| 144 | OR51F1 | na | 10994 | 0.058 | 0.1627 | No |
| 145 | OR2T12 | na | 11049 | 0.058 | 0.1620 | No |
| 146 | OR10R2 | na | 11070 | 0.057 | 0.1623 | No |
| 147 | OR13C2 | na | 11190 | 0.056 | 0.1597 | No |
| 148 | OR5M11 | na | 11283 | 0.055 | 0.1579 | No |
| 149 | OR6S1 | na | 11375 | 0.054 | 0.1561 | No |
| 150 | OR5M9 | na | 11406 | 0.053 | 0.1560 | No |
| 151 | CNGA4 | na | 11440 | 0.053 | 0.1559 | No |
| 152 | CNGB1 | na | 11630 | 0.051 | 0.1512 | No |
| 153 | OR1N2 | na | 11646 | 0.051 | 0.1515 | No |
| 154 | OR2D3 | na | 11653 | 0.050 | 0.1522 | No |
| 155 | OR51V1 | na | 11842 | 0.049 | 0.1474 | No |
| 156 | CALM2 | na | 11915 | 0.048 | 0.1461 | No |
| 157 | OR2S2 | na | 12155 | 0.046 | 0.1398 | No |
| 158 | OR11H4 | na | 12288 | 0.044 | 0.1367 | No |
| 159 | OR51A4 | na | 12384 | 0.044 | 0.1346 | No |
| 160 | OR2L13 | na | 12461 | 0.043 | 0.1331 | No |
| 161 | OR1L6 | na | 12500 | 0.042 | 0.1326 | No |
| 162 | OR52D1 | na | 12587 | 0.042 | 0.1308 | No |
| 163 | OR2AK2 | na | 12601 | 0.042 | 0.1310 | No |
| 164 | OR6K2 | na | 12685 | 0.041 | 0.1292 | No |
| 165 | OR10A5 | na | 12803 | 0.040 | 0.1265 | No |
| 166 | OR4X1 | na | 13109 | 0.037 | 0.1182 | No |
| 167 | OR4N5 | na | 13151 | 0.037 | 0.1175 | No |
| 168 | OR2AG2 | na | 13182 | 0.037 | 0.1172 | No |
| 169 | OR7D2 | na | 13471 | 0.034 | 0.1094 | No |
| 170 | CAMK2G | na | 13529 | 0.034 | 0.1083 | No |
| 171 | OR4K5 | na | 13799 | 0.032 | 0.1009 | No |
| 172 | OR4K1 | na | 14100 | 0.029 | 0.0926 | No |
| 173 | OR6F1 | na | 14200 | 0.029 | 0.0902 | No |
| 174 | OR4K2 | na | 14424 | 0.027 | 0.0841 | No |
| 175 | OR6B1 | na | 14558 | 0.026 | 0.0807 | No |
| 176 | OR4S1 | na | 14699 | 0.025 | 0.0770 | No |
| 177 | OR52E4 | na | 14838 | 0.024 | 0.0733 | No |
| 178 | OR8A1 | na | 14979 | 0.023 | 0.0696 | No |
| 179 | OR6B3 | na | 15085 | 0.022 | 0.0669 | No |
| 180 | OR2V2 | na | 15209 | 0.021 | 0.0637 | No |
| 181 | OR51E2 | na | 15358 | 0.020 | 0.0597 | No |
| 182 | OR56B1 | na | 15361 | 0.020 | 0.0599 | No |
| 183 | OR8I2 | na | 15430 | 0.020 | 0.0582 | No |
| 184 | OR51L1 | na | 15560 | 0.019 | 0.0548 | No |
| 185 | PRKX | na | 15799 | 0.017 | 0.0481 | No |
| 186 | CALM1 | na | 15870 | 0.017 | 0.0463 | No |
| 187 | CLCA4 | na | 15962 | 0.016 | 0.0439 | No |
| 188 | OR2F1 | na | 16038 | 0.016 | 0.0420 | No |
| 189 | OR9Q2 | na | 16061 | 0.015 | 0.0416 | No |
| 190 | OR52J3 | na | 16113 | 0.015 | 0.0404 | No |
| 191 | OR52I2 | na | 16766 | 0.011 | 0.0215 | No |
| 192 | OR2B3 | na | 16871 | 0.010 | 0.0187 | No |
| 193 | OR2G3 | na | 16979 | 0.009 | 0.0157 | No |
| 194 | OR52N2 | na | 17166 | 0.008 | 0.0104 | No |
| 195 | OR1S1 | na | 17184 | 0.008 | 0.0101 | No |
| 196 | OR10A6 | na | 17210 | 0.008 | 0.0095 | No |
| 197 | OR10AD1 | na | 17304 | 0.007 | 0.0069 | No |
| 198 | PDE1C | na | 17355 | 0.007 | 0.0055 | No |
| 199 | OR10H2 | na | 17413 | 0.007 | 0.0040 | No |
| 200 | OR10H5 | na | 17547 | 0.006 | 0.0002 | No |
| 201 | OR1Q1 | na | 17566 | 0.006 | -0.0002 | No |
| 202 | GUCY2D | na | 17892 | 0.003 | -0.0096 | No |
| 203 | OR51B6 | na | 18010 | 0.003 | -0.0130 | No |
| 204 | OR8G1 | na | 18344 | 0.000 | -0.0227 | No |
| 205 | OR8D4 | na | 18483 | -0.001 | -0.0267 | No |
| 206 | ARRB2 | na | 18679 | -0.002 | -0.0323 | No |
| 207 | OR5AK2 | na | 18762 | -0.003 | -0.0347 | No |
| 208 | OR56A3 | na | 18781 | -0.003 | -0.0352 | No |
| 209 | CLCA2 | na | 19031 | -0.005 | -0.0423 | No |
| 210 | OR10Z1 | na | 19276 | -0.007 | -0.0493 | No |
| 211 | OR1D2 | na | 19509 | -0.009 | -0.0560 | No |
| 212 | OR4C12 | na | 19624 | -0.010 | -0.0591 | No |
| 213 | OR6T1 | na | 20281 | -0.015 | -0.0780 | No |
| 214 | OR7G2 | na | 20354 | -0.016 | -0.0798 | No |
| 215 | CALM3 | na | 20684 | -0.019 | -0.0891 | No |
| 216 | PRKACA | na | 20814 | -0.020 | -0.0925 | No |
| 217 | OR2K2 | na | 20906 | -0.021 | -0.0949 | No |
| 218 | OR51T1 | na | 20983 | -0.022 | -0.0967 | No |
| 219 | OR5J2 | na | 21017 | -0.022 | -0.0973 | No |
| 220 | OR9G4 | na | 21426 | -0.026 | -0.1088 | No |
| 221 | OR2D2 | na | 21690 | -0.029 | -0.1160 | No |
| 222 | OR2Z1 | na | 21817 | -0.030 | -0.1192 | No |
| 223 | OR4N2 | na | 21961 | -0.032 | -0.1229 | No |
| 224 | OR5T2 | na | 22010 | -0.032 | -0.1238 | No |
| 225 | OR2H1 | na | 22094 | -0.033 | -0.1257 | No |
| 226 | OR14J1 | na | 22137 | -0.033 | -0.1264 | No |
| 227 | OR5V1 | na | 22255 | -0.035 | -0.1293 | No |
| 228 | OR6C4 | na | 22581 | -0.038 | -0.1381 | No |
| 229 | OR1J1 | na | 22805 | -0.041 | -0.1440 | No |
| 230 | OR2B11 | na | 23023 | -0.043 | -0.1496 | No |
| 231 | ADCY3 | na | 23170 | -0.045 | -0.1532 | No |
| 232 | OR51B5 | na | 23357 | -0.048 | -0.1578 | No |
| 233 | OR52A1 | na | 23855 | -0.054 | -0.1715 | No |
| 234 | OR2AE1 | na | 24036 | -0.057 | -0.1758 | No |
| 235 | OR51B4 | na | 24258 | -0.060 | -0.1813 | No |
| 236 | OR11A1 | na | 24429 | -0.062 | -0.1853 | No |
| 237 | OR52W1 | na | 24597 | -0.065 | -0.1892 | No |
| 238 | OR4X2 | na | 24610 | -0.065 | -0.1885 | No |
| 239 | OR10G4 | na | 24656 | -0.066 | -0.1888 | No |
| 240 | OR2A42 | na | 24966 | -0.070 | -0.1967 | No |
| 241 | OR52K1 | na | 25350 | -0.076 | -0.2067 | No |
| 242 | OR2A7 | na | 25442 | -0.077 | -0.2081 | No |
| 243 | OR1J4 | na | 25541 | -0.079 | -0.2097 | No |
| 244 | OR5I1 | na | 25581 | -0.080 | -0.2096 | No |
| 245 | OR2M5 | na | 25716 | -0.082 | -0.2122 | No |
| 246 | OR4F15 | na | 25835 | -0.084 | -0.2144 | No |
| 247 | OR4D6 | na | 26091 | -0.089 | -0.2204 | No |
| 248 | OR2H2 | na | 26127 | -0.090 | -0.2200 | No |
| 249 | OR1A1 | na | 26269 | -0.093 | -0.2227 | No |
| 250 | OR4E2 | na | 26273 | -0.093 | -0.2213 | No |
| 251 | OR51G1 | na | 26648 | -0.100 | -0.2306 | No |
| 252 | OR1I1 | na | 26727 | -0.102 | -0.2313 | No |
| 253 | PRKACB | na | 26730 | -0.102 | -0.2298 | No |
| 254 | GUCA1C | na | 26793 | -0.103 | -0.2300 | No |
| 255 | OR10G9 | na | 26799 | -0.103 | -0.2285 | No |
| 256 | OR1C1 | na | 26808 | -0.104 | -0.2272 | No |
| 257 | OR3A3 | na | 26834 | -0.104 | -0.2263 | No |
| 258 | OR2L8 | na | 26899 | -0.105 | -0.2265 | No |
| 259 | OR2J2 | na | 26952 | -0.107 | -0.2263 | No |
| 260 | OR8K5 | na | 27104 | -0.110 | -0.2290 | No |
| 261 | OR4A15 | na | 27176 | -0.112 | -0.2294 | No |
| 262 | GUCA1B | na | 27218 | -0.113 | -0.2288 | No |
| 263 | GNAL | na | 27327 | -0.115 | -0.2302 | No |
| 264 | OR1M1 | na | 27328 | -0.115 | -0.2284 | No |
| 265 | OR1J2 | na | 27517 | -0.119 | -0.2320 | No |
| 266 | OR2F2 | na | 27672 | -0.123 | -0.2346 | No |
| 267 | GUCA1A | na | 27735 | -0.125 | -0.2344 | No |
| 268 | OR2M2 | na | 27774 | -0.126 | -0.2336 | No |
| 269 | OR51S1 | na | 27817 | -0.127 | -0.2328 | No |
| 270 | OR5P2 | na | 27923 | -0.129 | -0.2339 | No |
| 271 | OR1F1 | na | 27963 | -0.130 | -0.2330 | No |
| 272 | OR10K1 | na | 28032 | -0.132 | -0.2329 | No |
| 273 | OR13J1 | na | 28033 | -0.132 | -0.2309 | No |
| 274 | OR10C1 | na | 28094 | -0.133 | -0.2305 | No |
| 275 | PRKACG | na | 28125 | -0.134 | -0.2293 | No |
| 276 | OR5M10 | na | 28252 | -0.137 | -0.2309 | No |
| 277 | OR5L1 | na | 28347 | -0.139 | -0.2314 | No |
| 278 | OR7E24 | na | 28387 | -0.140 | -0.2304 | No |
| 279 | OR4K13 | na | 28534 | -0.144 | -0.2324 | No |
| 280 | OR6C68 | na | 28539 | -0.145 | -0.2303 | No |
| 281 | OR1E1 | na | 28651 | -0.148 | -0.2312 | No |
| 282 | OR10G2 | na | 28956 | -0.156 | -0.2376 | No |
| 283 | OR5K1 | na | 29047 | -0.158 | -0.2378 | No |
| 284 | OR13G1 | na | 29161 | -0.162 | -0.2386 | No |
| 285 | OR5AU1 | na | 29218 | -0.163 | -0.2377 | No |
| 286 | OR52L1 | na | 29295 | -0.166 | -0.2373 | No |
| 287 | OR4C13 | na | 29341 | -0.167 | -0.2360 | No |
| 288 | OR4D2 | na | 29468 | -0.171 | -0.2370 | No |
| 289 | OR2B6 | na | 29535 | -0.173 | -0.2363 | No |
| 290 | OR9G1 | na | 29593 | -0.176 | -0.2352 | No |
| 291 | OR2J3 | na | 29674 | -0.178 | -0.2348 | No |
| 292 | OR52A5 | na | 29859 | -0.184 | -0.2372 | No |
| 293 | OR5A1 | na | 29892 | -0.185 | -0.2353 | No |
| 294 | OR4D9 | na | 29981 | -0.188 | -0.2349 | No |
| 295 | OR2T4 | na | 30041 | -0.189 | -0.2337 | No |
| 296 | OR51B2 | na | 30093 | -0.191 | -0.2322 | No |
| 297 | OR13C8 | na | 30111 | -0.192 | -0.2297 | No |
| 298 | OR52M1 | na | 30176 | -0.194 | -0.2286 | No |
| 299 | OR11L1 | na | 30288 | -0.197 | -0.2288 | No |
| 300 | OR8B8 | na | 30350 | -0.199 | -0.2274 | No |
| 301 | OR9A2 | na | 30406 | -0.201 | -0.2259 | No |
| 302 | OR2T5 | na | 30490 | -0.203 | -0.2252 | No |
| 303 | OR10Q1 | na | 30508 | -0.204 | -0.2225 | No |
| 304 | OR7C2 | na | 30524 | -0.205 | -0.2198 | No |
| 305 | OR4D10 | na | 30590 | -0.207 | -0.2184 | No |
| 306 | OR7G3 | na | 30615 | -0.208 | -0.2159 | No |
| 307 | OR5B12 | na | 30669 | -0.210 | -0.2142 | No |
| 308 | PDC | na | 30728 | -0.212 | -0.2126 | No |
| 309 | OR51G2 | na | 30869 | -0.216 | -0.2133 | No |
| 310 | OR51A2 | na | 30921 | -0.218 | -0.2114 | No |
| 311 | OR6C3 | na | 30932 | -0.218 | -0.2083 | No |
| 312 | OR2M3 | na | 30958 | -0.219 | -0.2056 | No |
| 313 | OR52B4 | na | 31035 | -0.222 | -0.2044 | No |
| 314 | OR4B1 | na | 31066 | -0.223 | -0.2018 | No |
| 315 | OR6C76 | na | 31142 | -0.226 | -0.2005 | No |
| 316 | PRKG1 | na | 31202 | -0.229 | -0.1986 | No |
| 317 | OR1G1 | na | 31271 | -0.231 | -0.1970 | No |
| 318 | OR5A2 | na | 31291 | -0.232 | -0.1940 | No |
| 319 | OR2T27 | na | 31344 | -0.234 | -0.1918 | No |
| 320 | OR5AP2 | na | 31365 | -0.235 | -0.1888 | No |
| 321 | OR2G6 | na | 31477 | -0.239 | -0.1883 | No |
| 322 | OR10J3 | na | 31487 | -0.240 | -0.1848 | No |
| 323 | OR6C70 | na | 31531 | -0.241 | -0.1823 | No |
| 324 | OR2C1 | na | 31655 | -0.247 | -0.1821 | No |
| 325 | OR7G1 | na | 31667 | -0.247 | -0.1785 | No |
| 326 | OR8H1 | na | 31683 | -0.248 | -0.1751 | No |
| 327 | OR1L4 | na | 31727 | -0.250 | -0.1725 | No |
| 328 | OR10G7 | na | 31745 | -0.251 | -0.1691 | No |
| 329 | OR5AC2 | na | 31791 | -0.254 | -0.1664 | No |
| 330 | OR52N4 | na | 31877 | -0.257 | -0.1649 | No |
| 331 | OR4L1 | na | 32043 | -0.266 | -0.1656 | No |
| 332 | OR5M3 | na | 32052 | -0.267 | -0.1617 | No |
| 333 | OR2A5 | na | 32226 | -0.278 | -0.1624 | No |
| 334 | OR8J3 | na | 32358 | -0.285 | -0.1618 | No |
| 335 | OR4C15 | na | 32419 | -0.288 | -0.1590 | No |
| 336 | OR6Q1 | na | 32443 | -0.290 | -0.1552 | No |
| 337 | OR56A4 | na | 32494 | -0.293 | -0.1521 | No |
| 338 | OR1S2 | na | 32558 | -0.298 | -0.1493 | No |
| 339 | OR2T33 | na | 32704 | -0.306 | -0.1488 | No |
| 340 | OR2W1 | na | 32722 | -0.307 | -0.1445 | No |
| 341 | OR13A1 | na | 32843 | -0.317 | -0.1430 | No |
| 342 | CAMK2D | na | 32964 | -0.326 | -0.1415 | No |
| 343 | OR5H6 | na | 32979 | -0.327 | -0.1368 | No |
| 344 | OR8K3 | na | 32986 | -0.328 | -0.1319 | No |
| 345 | OR6X1 | na | 33100 | -0.337 | -0.1299 | No |
| 346 | OR4C16 | na | 33102 | -0.337 | -0.1247 | No |
| 347 | OR7A10 | na | 33155 | -0.341 | -0.1209 | No |
| 348 | OR51I2 | na | 33229 | -0.346 | -0.1177 | No |
| 349 | OR8U1 | na | 33278 | -0.351 | -0.1136 | No |
| 350 | OR8U8 | na | 33294 | -0.352 | -0.1086 | No |
| 351 | OR13C5 | na | 33301 | -0.353 | -0.1033 | No |
| 352 | OR8H3 | na | 33365 | -0.358 | -0.0995 | No |
| 353 | OR6Y1 | na | 33401 | -0.361 | -0.0949 | No |
| 354 | OR7D4 | na | 33417 | -0.363 | -0.0897 | No |
| 355 | OR51M1 | na | 33590 | -0.378 | -0.0889 | No |
| 356 | OR51I1 | na | 33638 | -0.382 | -0.0843 | No |
| 357 | OR52E2 | na | 33654 | -0.384 | -0.0788 | No |
| 358 | OR4A5 | na | 33708 | -0.391 | -0.0742 | No |
| 359 | OR4M1 | na | 33720 | -0.394 | -0.0684 | No |
| 360 | OR1L3 | na | 33911 | -0.418 | -0.0674 | No |
| 361 | OR11H6 | na | 33943 | -0.422 | -0.0618 | No |
| 362 | OR4K14 | na | 33952 | -0.423 | -0.0554 | No |
| 363 | OR51D1 | na | 34045 | -0.436 | -0.0513 | No |
| 364 | OR4C45 | na | 34049 | -0.437 | -0.0446 | No |
| 365 | OR4D11 | na | 34109 | -0.444 | -0.0395 | No |
| 366 | OR4D5 | na | 34264 | -0.476 | -0.0365 | No |
| 367 | OR13H1 | na | 34266 | -0.476 | -0.0292 | No |
| 368 | OR52R1 | na | 34302 | -0.486 | -0.0226 | No |
| 369 | OR2Y1 | na | 34309 | -0.488 | -0.0152 | No |
| 370 | OR10J5 | na | 34469 | -0.540 | -0.0115 | No |
| 371 | OR2A25 | na | 34574 | -0.600 | -0.0052 | No |
| 372 | OR8H2 | na | 34591 | -0.619 | 0.0040 | No |
Table: GSEA details [plain text format]

  

Fig 2: KEGG\_OLFACTORY\_TRANSDUCTION      
 Blue-Pink O' Gram in the Space of the Analyzed GeneSet

  

Fig 3: KEGG\_OLFACTORY\_TRANSDUCTION: Random ES distribution      
 Gene set null distribution of ES for **KEGG\_OLFACTORY\_TRANSDUCTION**
